# Quantitative determination, principal component analysis and discriminant analysis of eight marker compounds in crude and sweated Dipsaci Radix by HPLC-DAD

**DOI:** 10.1080/13880209.2017.1297469

**Published:** 2017-10-02

**Authors:** Weifeng Du, Xiaoning Li, Ying Yang, Xianke Yue, Dongjing Jiang, Weihong Ge, Baochang Cai

**Affiliations:** a Research Center of TCM Processing Technology, Zhejiang Chinese Medical University, Hangzhou, China;; b Jiangsu Key Laboratory of Chinese Medicine Processing, Nanjing University of Chinese Medicine, Nanjing, China

**Keywords:** Primary processing, quality control, PCA, DA

## Abstract

**Context:** Dipsaci Radix is derived from the dry root of *Dipsacus asper* Wall.ex Henry (Dipsacaceae). It has attracted increasing attention as one of the most popular and precious herbal medicines in clinical use.

**Objective:** To develop a HPLC-DAD method for quantitative analysis and quality control of eight active components in crude and sweated Dipsaci Radix.

**Materials and methods:** The eight components in Dipsaci Radix were analyzed by HPLC-DAD on an Agilent Eclipse XDB-C18 column within a gradient elution of acetonitrile and 0.05% formic acid aqueous solution. ESI-MS spectra were acquired on a triple quadrupole mass spectrometer. Validation was performed in order to demonstrate linearity, precision, repeatability, stability, and accuracy of the method. The results were processed with principal component analysis (PCA) and discriminant analysis (DA).

**Results:** The eight components showed good linearity (*R*^2^ > 0.9991) in the ranges of 60.40–1208.00, 151.00–3020.00, 3.06–61.20, 30.76–615.20, 5.13–102.60, 10.17–203.40, 10.20–204.00, and 151.60–3032.00 mg/mL, respectively. The overall recoveries were in the range of 99.03–102.38%, with RSDs ranging from 1.89% to 4.05%. Through PCA, the degree of importance of the eight components in sequence was CA > AVI > IA > LA > LN > IC > IB > CaA. The crude and sweated Dipsaci Radix were distinguished obviously by DA.

**Discussion and conclusion:** The method, using HPLC-DAD analysis in combination with PCA and DA, could provide a more comprehensive and quantitative chemical pattern recognition and quality evaluation to crude and sweated Dipsaci Radix.

## Introduction

Dipsaci Radix, derived from the dry roots of *Dipsacus asper* Wall. ex Henry (Dipsacaceae) (Chinese Pharmacopoeia Commission 2015), is a traditional herbal medicine with a long history for the treatment of bone fractures and low back pain in China (Wong et al. 2007; Peng et al. 2010; Niu et al. 2012, 2015). Sweating, which used to be a traditional method of processing fresh Dipsaci Radix in the production area for drying (Jin et al. 2010), is no longer commonly adopted. So the effect of the sweating to Dipsaci Radix should be studied based on the perspective of the chemical compositions.

In the last decades, the phytochemistry of Dipsaci Radix has been extensively investigated, and the results indicate that loganic acid, chlorogenic acid, caffeic acid, loganin, isochlorogenic acid A, isochlorogenic acid B, isochlorogenic acid C, and asperosaponin VI are the main active components (Wei et al. 2011; Liu et al. 2012; Du et al. 2014; Ling et al. 2014). Pharmacological studies on the components showed that they all had various biological activities. Loganic acid has anti-inflammatory activity due to COX inhibition (Ramírez-Cisneros et al. 2015), and could be used to reduce intraocular pressure (Szumny et al. 2015). Loganin and chlorogenic acid also have anti-inflammatory activity (Lou et al. 2015; Kim et al. 2015a). Caffeic acid inhibits lipid peroxidation (Kim et al. 2015b). Isochlorogenic A, B, C have anti-inflammatory and antimicrobial effects (Chen et al. 2015). Asperosaponin VI has antioxidant activity (Song et al. 2014), and could promote bone cell proliferation (Niu et al. 2011).

To our knowledge, previously reported analytical methods were employed to rapidly separate and identify lots of components in Dipsaci Radix (Wei et al. 2011; Liu et al. 2012; Du et al. 2014; Ling et al. 2014), but to quantify only several components in crude Dipsaci Radix (Li et al. 2011; Liu et al. 2011; Fan et al. 2013; Du et al. 2013; Zhao et al. 2013; Fan et al. 2015; Wang et al. 2015; Zhang et al. 2015; Du et al. 2016). In this study, a high performance liquid chromatography with diode array detector (HPLC-DAD) method was developed to quantify eight major bioactive components simultaneously in both the crude and its processed products. The method is simple, quick, and cheap with good reproducibility. It offers a new method that could be employed for the quality control of not only crude Dipsaci Radix, but also its processed products.

## Materials and methods

### Chemicals and reagents

Acetonitrile of HPLC grade and methanol for analysis were provided by Tedia Co. (Fairfield, OH). Formic acid of reagent grade was purchased from Zhejiang SanYing Chemical Co., Ltd. (Zhejiang, China). Ultrapure water (0.45 μm) was prepared by using microporous membrane filter (Shanghai XingYa, China). All other chemicals and solvents used in this study were of analytical grade. The reference substances of loganic acid (LA, 93.8%), chlorogenic acid (CA, 96.6%), caffeic acid (CaA, 100.0%), loganin (LN, 99.2%), and asperosaponin VI (AVI, 93.5%) were purchased from National Institutes for Food and Drug Control. The isochlorogenic acid A (IA, 98.0%), isochlorogenic acid B (IB, 98.0%), isochlorogenic acid C (IC, 98.0%) were purchased from Chengdu Must Bio-technology Co., Ltd. (Sichuan, China).

### Plant material

Dipsaci Radix used in this study were collected from different origins (Table 1), the crude and sweated samples all were processed from the same batch of fresh herbs in the production area. The crude samples were produced after drying the fresh herbs directly by oven, and the sweated ones were produced after drying the fresh herbs pre-sweated to internal turning green. These herbal samples were authenticated by Professor Pingfan Lai (Zhejiang Chinese Medical University, Hangzhou, China). These specimens were preserved in the Research Center of TCM Processing Technology, Zhejiang Chinese Medical University.

**Table 1.** The origin and respective voucher number of Dipsaci Radix.

Samples	Origin	Voucher number
Crude drug	Sichuan-1	C20131209
	Sichuan-2	C20131209-1
	Sichuan-3	C20131209-2
	Sichuan-4	C20131209-3
	Sichuan-5	C20131006-3
	Hubei-1	C20131025
	Hubei-2	C20131006-4
	Hubei-3	C20131006-2
	Guizhou	C20131105-6
	Yunnan	C20131031
	Jiangxi	C20131006-5
Sweated sample	Sichuan-1	S20131209
	Sichuan-2	S20131209-1
	Sichuan-3	S20131209-2
	Sichuan-4	S20131209-3
	Sichuan-5	S20131006-3
	Hubei-1	S20131025
	Hubei-2	S20131006-4
	Hubei-3	S20131006-2
	Guizhou	S20131105-6
	Yunnan	S20131031
	Jiangxi	S20131006-5

### Apparatus and chromatographic conditions

The simultaneous determination was performed with a HPLC system Agilent 1200 (Agilent Technologies, Santa Clara, CA) consisting of quaternary pump (G1311A), auto sampler injector (G1329A), column oven (G1316A), and diode array detector (G1315D). Data were collected and processed by HPLC solution software (Santa Clara, CA). ESI-MS spectra were acquired on an Agilent 6410 triple quadrupole mass spectrometer (Agilent Technologies, Santa Clara, CA), and the electrospray ionization (ESI) conditions were set as follows: ion mode, negative; scan range, *m/z* 100–2000; dry gas, N_2_, dry gas flow, 10 L/min; dry heater, 300 °C; nebulizer pressure, 15 psi; capillary voltage, 4000 V; fragmentor voltage, 135 V; collision voltage, 25 eV. Separation of eight compounds was carried out on an Eclipse XDB-C18 analytical column (Agilent, Santa Clara, CA, 250 mm ×4.6 mm, 5.0 μm particle size). The mobile phase for chromatographic separation of (A) 0.05% formic acid aqueous solution (V/V) and (B) acetonitrile using a gradient elution of 2–6% B at 0–5 min, 6–10% B at 5–18 min, 10–20% B at 18–40 min, 20–25% B at 40–70 min, 25–35% B at 70–80 min, 35–60% B at 80–90 min, 60–70% B at 90–110 min, 70% B at 110–120 min. The flow rate was 1.0 mL/min, and the column temperature was maintained at 30 °C. The DAD detector was set at 212 nm, and the UV absorption spectra were recorded in the range of 210–400 nm.

### Preparation of standard solutions

Primary stock standard solutions of the eight compounds were prepared in methanol with a concentration of 1208.0 μg/mL for LA, 3020.0 μg/mL for CA, 61.2 μg/mL for CaA, 615.2 μg/mL for LN, 102.6 μg/mL for IB, 203.4 μg/mL for IA, 204.0 μg/mL for IC and 3032.0 μg/mL for AVI. The standard stock solution was further diluted with methanol to make10 different concentrations including 1, 3/4, 1/2, 2/5, 3/10, 1/4, 1/5, 3/20, 1/10 and 1/20 of the original concentration. The solutions were filtered through a polyvinylidene difluoride filter of 0.45 μm and stored at 4 °C.

### Preparation of sample solutions

The dried powder of Dipsaci Radix (0.500 g, 80 mesh) was accurately weighed and added into dark brown calibrated flasks (100 mL). Methanol (25 mL) was added, the weight was accurately measured, and the sample was sonicated for 30 min. The solution was weighed again, and the loss in weight was made up with methanol. The supernatants were filtered through a 0.45 μm membrane prior to injection.

### Calibration curves and limits of detection and quantification

The calibration curves were performed with 10 different concentrations in triplicate. The regression equations were established by plotting the peak area (*y*) versus concentration (*x*) of each analyte. The linearity was measured by correlation coefficient (*R*^2^) values. Limit of detection (LOD) and quantification (LOQ) were determined by injecting a series of standard solutions until the signal-to-noise ratio (S/N) for each compound was 3 for LOD and 10 for LOQ, respectively. The results were given in Table 2.

**Table 2.** Linear relation between peak area and concentration.

Analytes	Regression equation	Correlation Coefficient (*R^*2*^*)	Linear range (μg/mL)	LOD (μg/mL)	LOQ (μg/mL)
LA	*y* = 4.3712*x* − 39.286	0.9996	60.40–1208.00	0.15	0.50
CA	*y* = 1.9659*x* + 20.113	0.9998	151.00–3020.00	0.15	0.50
CaA	*y* = 31.195*x* + 40.929	0.9991	3.06–61.20	0.12	0.31
LN	*y* = 4.5192*x* − 1.2019	0.9998	30.76–615.20	0.12	0.31
I B	*y* = 32.669*x* + 0.4779	0.9998	5.13–102.60	0.15	0.51
IA	*y* = 16.765*x* + 30.678	0.9996	10.17–203.40	0.10	0.31
IC	*y* = 23.963*x* − 7.5264	0.9998	10.20–204.00	0.10	0.31
AVI	*y* = 1.7764*x* − 3.7019	0.9998	151.60–3032.00	0.15	0.50

### Precision, repeatability and stability

The intra- and inter-day precision was determined by analyzing calibration samples during a single day and on six different days, respectively. The intra-day variation was determined by analyzing the six replicates on the same day and inter-day variation was determined on six consecutive days. Overall intra- and inter-day variations were less than 1.77%.

To further evaluate the repeatability of the developed assay, Dipsaci Radix was analyzed in six replicates as described above. The contents of eight compounds were calculated from the corresponding calibration curves. The relative standard deviations (RSDs) were taken as measurements of repeatability. Stability was tested with Dipsaci Radix at room temperature and analyzed at 0, 2, 4, 8, 12, 24, and 48 h within 2 days, As a result, the RSDs of repeatability test and stability test were both less than 3.45%. The results were given in Tables 3 and 4.

**Table 3.** Precision of analytical results (*n = 6*).

		Intra-day	Inter-day
Analytes	Standard solution (μg/mL)	Precision (RSD/%)	Precision (RSD/%)
LA	120.80	0.71	1.24
	241.60	0.67	1.21
	604.00	0.62	1.18
CA	302.00	0.86	1.02
	604.00	0.52	1.43
	1510.00	0.50	0.94
CaA	6.12	1.10	1.77
	12.24	0.83	1.66
	30.60	0.52	1.32
LN	61.52	1.04	1.58
	123.04	0.85	1.53
	307.60	0.80	1.39
IB	10.26	0.86	1.22
	20.52	0.86	1.35
	51.30	0.51	1.18
IA	20.34	0.79	0.98
	40.68	0.62	1.58
	101.70	0.59	1.54
IC	20.40	0.61	1.52
	40.80	0.76	1.31
	102.00	0.77	0.98
AVI	303.20	0.53	1.17
	606.40	0.83	1.27
	1516.00	0.77	0.96

**Table 4.** Repeatability and stability of analytical results (*n = 6*).

	Repeatability (% ± SD, RSD/%)		Stability (% ± SD, RSD/%)	
Analytes	Crude drug	Sweated sample	Crude drug	Sweated sample
LA	0.86 ± 0.02, 2.02	0.71 ± 0.02, 2.73	0.87 ± 0.01, 1.21	0.72 ± 0.01, 1.29
CA	2.54 ± 0.04, 1.75	2.33 ± 0.05, 2.28	2.51 ± 0.04, 1.56	2.39 ± 0.04, 1.61
CaA	0.11 ± 0.00, 2.23	0.11 ± 0.00, 3.44	0.10 ± 0.00, 1.57	0.11 ± 0.00, 1.97
LN	0.57 ± 0.01, 1.90	0.53 ± 0.02, 3.45	0.57 ± 0.01, 2.15	0.54 ± 0.01, 1.82
IB	0.12 ± 0.00, 1.64	0.13 ± 0.00, 1.95	0.12 ± 0.00, 1.42	0.13 ± 0.00, 1.86
IA	0.59 ± 0.02, 2.60	0.58 ± 0.01, 2.55	0.59 ± 0.01, 1.05	0.58 ± 0.01, 1.43
IC	0.38 ± 0.01, 2.53	0.48 ± 0.02, 3.44	0.37 ± 0.01, 1.42	0.48 ± 0.00, 0.85
AVI	6.26 ± 0.15, 2.35	5.37 ± 0.08, 1.40	6.25 ± 0.06, 0.89	5.39 ± 0.07, 1.22

### Accuracy

Accuracy was determined by the recovery test. An appropriate amount of Dipsaci Radix powder was weighed and spiked with 80%, 100% and 120% of known amount of each standard compound. They were then treated and analyzed as described above. Each sample was analyzed in six replicates. The total amount of each analyte was calculated from the corresponding calibration curve. Mean recoveries of eight compounds were 99.03–102.38%. The results were given in Table 5.

**Table 5.** Recoveries of marker compounds through standard addition (*n = 6*).

Analytes	Original (mg ± SD)	Spiked (mg)	Determined (mg ± SD)	Recovery (% ± SD, RSD%)
LA	2.03 ± 0.00	1.60	3.66 ± 0.07	101.86 ± 4.12, 4.05
		2.00	4.05 ± 0.05	100.91 ± 2.75, 2.73
		2.40	4.46 ± 0.07	101.24 ± 2.84, 2.80
CA	5.93 ± 0.01	4.80	10.75 ± 0.14	100.39 ± 2.84, 2.83
		6.00	12.01 ± 0.23	101.43 ± 3.78, 3.73
		7.20	13.07 ± 0.21	99.22 ± 3.01, 3.03
CaA	0.25 ± 0.00	0.20	0.45 ± 0.01	100.47 ± 3.84, 3.82
		0.25	0.50 ± 0.01	100.38 ± 3.96, 3.94
		0.30	0.55 ± 0.01	99.76 ± 3.63, 3.64
LN	1.23 ± 0.00	0.96	2.21 ± 0.02	102.35 ± 1.94, 1.89
		1.20	2.42 ± 0.03	99.38 ± 2.57, 2.59
		1.44	2.70 ± 0.05	102.26 ± 3.33, 3.26
IB	0.27 ± 0.00	0.20	0.47 ± 0.01	99.91 ± 2.70, 2.70
		0.25	0.53 ± 0.01	101.26 ± 2.05, 2.02
		0.30	0.57 ± 0.01	99.94 ± 3.95, 3.95
IA	1.34 ± 0.00	1.08	2.45 ± 0.03	102.18 ± 2.63, 2.57
		1.35	2.68 ± 0.04	99.03 ± 2.68, 2.71
		1.62	3.00 ± 0.04	102.38 ± 2.17, 2.12
IC	0.87 ± 0.00	0.70	1.57 ± 0.02	99.84 ± 3.05, 3.04
		0.85	1.71 ± 0.02	99.28 ± 2.19, 2.21
		1.02	1.91 ± 0.03	102.01 ± 3.17, 3.10
AVI	14.43 ± 0.02	11.60	26.23 ± 0.24	101.72 ± 2.17, 2.14
		14.50	29.06 ± 0.48	101.29 ± 2.81, 2.77
		17.40	32.21 ± 0.54	102.21 ± 3.16, 3.09

Recovery (%)

 = (Amount_found_ − Amount_original_)/Amount_spiked_ × 100%

## Results and discussion

### Optimization of chromatographic conditions

**Figure 1.**
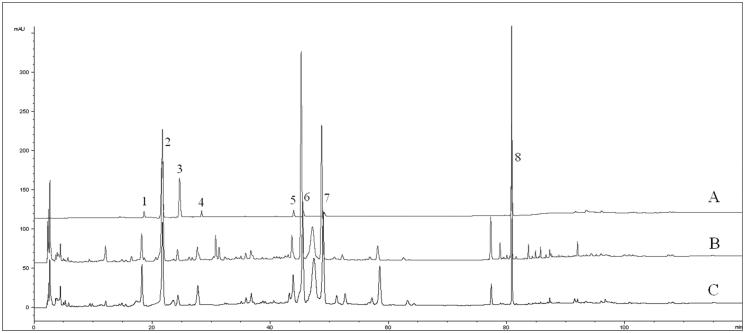
HPLC chromatogram of standard mixture (A), crude drug (B) and sweated sample (C): (1) LA; (2) CA; (3) CaA; (4) LN; (5) IB; (6) IA; (7) IC and (8) AVI.

High performance liquid chromatography conditions including column type, column temperature, flow rate and mobile phases were assessed to accomplish the simultaneous separation of the eight analytes. The theoretical plate, symmetry factor and resolution were evaluated. To evaluate the suitability, three different columns, Zorbax Extend-C18, Eclipse XDB-C18 and Inertsil ODS-SP were compared with regard to the three analytical factors. As a result, the Eclipse XDB-C18 was the best for separation. Furthermore, other chromatographic variables were also optimized on the Eclipse XDB-C18 column, including mobile phases (water-methanol, water-acetonitrile and aqueous formic acid-acetonitrile), the column temperatures (20, 25, and 30 °C) and the flow rates (0.8 and 1.0 mL/min). Eventually, the optimal separation was achieved on an Agilent Eclipse XDB-C18 (250 mm × 4.6 mm, 5.0 μm) at a column temperature of 30 °C with a flow rate of 1.0 mL/min. The HPLC chromatograms were shown in Figure 1.

### Qualitative analysis of eight compounds

**Figure 2.**
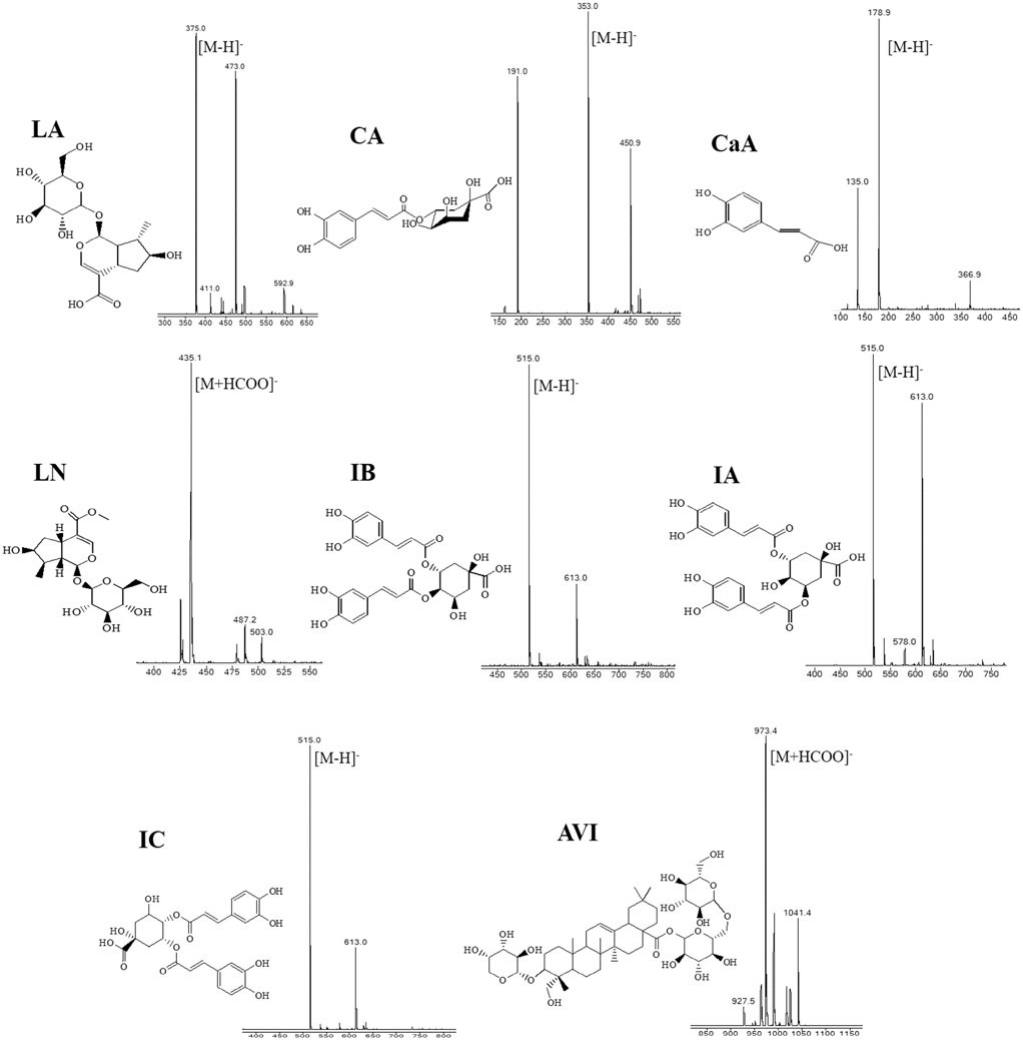
ESI-MS spectra of eight compounds.

The developed HPLC method was applied to identify eight components (LA, CA, CaA, LN, IB, IA, IC and AVI) in crude and sweated Dipsaci Radix samples from different production area. The chromatographic peaks of eight components were confirmed by comparing their retention time, UV and MS spectrum with those of the corresponding reference compounds. The retention time for LA, CA, CaA, LN, IB, IA, IC and AVI were 18.37 ± 0.11, 21.94 ± 0.09, 24.48 ± 0.04, 27.87 ± 0.09, 44.21 ± 0.02, 45.58 ± 0.05, 49.05 ± 0.04, and 81.12 ± 0.05 min, respectively. The maximum absorption wavelengths of LA, CA, CaA, LN, IB, IA, IC and AVI were 240, 330, 330, 240, 330, 330, 330 and 212 nm, respectively. As depicted in Figure 2, the ESI-MS spectra gave quasimolecular ions of LA (*m/z* 375.0 [M–H]^-^) (Papalexandrou et al. 2003), CA (*m/z* 353.0 [M–H]^-^) (Kowalczyk & Krzyzanowska 1999), CaA (*m/z* 178.9 [M–H]^-^) (Tian 2006), LN (*m/z* 435.1 [M + HCOO]^-^) (Papalexandrou et al. 2003), IB (*m/z* 515.0 [M–H]^-^), IA(*m/z* 515.0 [M–H]^-^), IC (*m/z* 515.0 [M–H]^-^) (Hung et al. 2005) and AVI (*m/z* 973.4 [M + HCOO]^-^) (Kouno et al. 1990).

### Quantitative analysis of eight compounds

The established HPLC method was successfully applied to the simultaneous determination of eight compounds in crude and sweated Dipsaci Radix samples. All of the contents were summarized in Table 6.

**Table 6.** Contents of eight components in Dipsaci Radix (% ± SD, *n* = 3).

Suppliers	Samples	LA	CA	CaA	LN	IB	IA	IC	AVI
Sichuan-1	Crude drug	1.10 ± 0.03	8.28 ± 0.21	0.01 ± 0.00	0.61 ± 0.03	0.09 ± 0.00	0.92 ± 0.03	0.48 ± 0.02	15.19 ± 0.34
	Sweated sample	0.98 ± 0.04	2.49 ± 0.11	0.05 ± 0.00	0.45 ± 0.02	0.14 ± 0.01	0.70 ± 0.03	0.59 ± 0.02	2.50 ± 0.12
	change rate	−10.91%	−69.93%	+400.00%	−26.23%	+55.56%	−23.91%	+22.92%	−83.54%
Sichuan-2	Crude drug	1.10 ± 0.04	8.28 ± 0.22	0.01 ± 0.00	0.61 ± 0.03	0.09 ± 0.00	0.92 ± 0.04	0.48 ± 0.02	15.19 ± 0.35
	Sweated sample	0.94 ± 0.03	4.11 ± 0.21	0.09 ± 0.00	0.51 ± 0.02	0.18 ± 0.01	0.68 ± 0.03	0.80 ± 0.02	2.31 ± 0.11
	change rate	−14.55%	−50.36%	+800.00%	−16.39%	+100.00%	−26.09%	+66.67%	−84.79%
Sichuan-3	Crude drug	1.10 ± 0.03	8.28 ± 0.25	0.01 ± 0.00	0.61 ± 0.02	0.09 ± 0.00	0.92 ± 0.03	0.48 ± 0.01	15.19 ± 0.42
	Sweated sample	0.93 ± 0.02	2.99 ± 0.12	0.07 ± 0.00	0.43 ± 0.02	0.16 ± 0.01	0.55 ± 0.02	0.55 ± 0.02	1.95 ± 0.09
	change rate	15.45%	−63.89%	+600.00%	−29.51%	+77.78%	−40.22%	+14.58%	−87.16%
Sichuan-4	Crude drug	1.10 ± 0.04	8.28 ± 0.26	0.01 ± 0.00	0.61 ± 0.02	0.09 ± 0.00	0.92 ± 0.04	0.48 ± 0.02	15.19 ± 0.38
	Sweated sample	0.96 ± 0.03	2.61 ± 0.13	0.13 ± 0.01	0.49 ± 0.02	0.12 ± 0.01	0.59 ± 0.02	0.55 ± 0.02	1.94 ± 0.06
	change rate	−12.73%	−68.48%	+1200.00%	−29.51%	+77.78%	−35.87%	+14.58%	−87.23%
Sichuan-5	Crude drug	0.89 ± 0.04	7.62 ± 0.08	0.01 ± 0.01	0.54 ± 0.02	0.08 ± 0.01	0.79 ± 0.02	0.38 ± 0.01	6.33 ± 0.21
	Sweated sample	0.68 ± 0.03	2.29 ± 0.06	0.11 ± 0.01	0.42 ± 0.01	0.13 ± 0.01	0.58 ± 0.02	0.46 ± 0.02	5.31 ± 0.17
	change rate	−23.60%	−69.95%	+1000.00%	−22.22%	+62.50%	−26.58%	+14.58%	−16.11%
Hubei-1	Crude drug	0.86 ± 0.04	2.57 ± 0.08	0.16 ± 0.00	0.57 ± 0.02	0.11 ± 0.01	0.67 ± 0.03	0.64 ± 0.01	1.61 ± 0.06
	Sweated sample	0.68 ± 0.03	2.23 ± 0.07	0.18 ± 0.01	0.54 ± 0.01	0.13 ± 0.01	0.61 ± 0.03	0.78 ± 0.03	1.36 ± 0.04
	change rate	−20.93%	−13.23%	+12.50%	−5.26%	+18.18%	−8.96%	+17.95%	−15.53%
Hubei-2	Crude drug	0.83 ± 0.03	6.85 ± 0.24	0.02 ± 0.00	0.51 ± 0.01	0.08 ± 0.00	0.73 ± 0.03	0.63 ± 0.03	4.99 ± 0.21
	Sweated sample	0.54 ± 0.02	5.00 ± 0.15	0.03 ± 0.00	0.39 ± 0.01	0.09 ± 0.00	0.60 ± 0.02	0.73 ± 0.03	3.58 ± 0.13
	change rate	−34.94%	−27.01%	+50.00%	−23.53%	+12.50%	−17.81%	+15.87%	−28.26%
Hubei-3	Crude drug	1.01 ± 0.04	4.38 ± 0.09	0.10 ± 0.00	0.48 ± 0.01	0.12 ± 0.01	0.88 ± 0.03	0.48 ± 0.02	9.59 ± 0.27
	Sweated sample	0.81 ± 0.03	4.25 ± 0.18	0.11 ± 0.01	0.47 ± 0.01	0.13 ± 0.01	0.81 ± 0.03	0.58 ± 0.02	9.31 ± 0.32
	change rate	−19.80%	−2.97%	+10.00%	−2.08%	+8.33%	−7.95%	+20.83%	−2.92%
Jiangxi	Crude drug	0.81 ± 0.04	6.79 ± 0.21	0.03 ± 0.00	0.49 ± 0.02	0.08 ± 0.00	0.74 ± 0.02	0.73 ± 0.03	4.78 ± 0.15
	Sweated sample	0.53 ± 0.02	4.54 ± 0.17	0.04 ± 0.00	0.38 ± 0.01	0.10 ± 0.00	0.63 ± 0.02	0.78 ± 0.03	3.43 ± 0.12
	change rate	−34.57%	−33.14%	+33.33%	−22.45%	+25.00%	−14.86%	+14.58%	−28.24%
Guizhou	Crude drug	0.71 ± 0.03	2.02 ± 0.07	0.03 ± 0.00	0.29 ± 0.01	0.08 ± 0.00	0.77 ± 0.02	0.26 ± 0.01	4.74 ± 0.22
	Sweated sample	0.50 ± 0.02	1.67 ± 0.06	0.04 ± 0.00	0.21 ± 0.01	0.09 ± 0.00	0.75 ± 0.03	0.27 ± 0.01	4.16 ± 0.15
	change rate	−29.58%	−17.33%	+33.33%	−27.59%	+12.50%	−2.60%	+3.85%	−12.24%
Yunnan	Crude drug	0.79 ± 0.03	2.54 ± 0.08	0.06 ± 0.00	0.48 ± 0.02	0.06 ± 0.00	0.66 ± 0.02	0.35 ± 0.01	2.83 ± 0.12
	Sweated sample	0.66 ± 0.02	1.89 ± 0.07	0.07 ± 0.00	0.38 ± 0.01	0.07 ± 0.00	0.48 ± 0.02	0.44 ± 0.02	2.14 ± 0.08
	change rate	−16.46%	−25.59%	+16.67%	−20.83%	+16.67%	−27.27%	+25.71%	−24.38%

“−”decreased; “+” increased.

The results showed that crude drug from different origins contained LA 0.71–1.10%, CA 2.02–8.28%, CaA 0.01–0.16%, LN 0.29–0.61%, IB 0.08–0.13%, IA 0.59–0.92%, IC 0.26–0.73% and AVI 1.61–15.19%, and the sweated samples contained LA 0.50–0.98%, CA 1.67–5.00%, CaA 0.03–0.18%, LN 0.21–0.55%, IB 0.09–0.18%, IA 0.48–0.81%, IC 0.27–0.80% and AVI 1.36–9.31%. A number of factors might contribute to the variation of contents, such as plant origin, genetic variation, growth circumstance, processing, storage conditions and so on. Therefore, quality control for Dipsaci Radix pieces was very necessary. Corresponding batches of crude and sweated samples, the contents of eight components varied significantly, especially the samples from Sichuan. This suggested that the ‘sweating’ influenced the contents of these compounds. Through the ‘sweating’ processing, the contents of LA, CA, LN, IA and AVI decreased, the change rate were 10.91–34.94%, 2.97–69.95%, 2.08–29.51%, 2.60–40.22%, 2.92–87.23%, respectively; those of CaA, IB and IC increased, the change rate were 10–1200%, 8.33–100%, 3.85–66.67%, respectively. For instance, Dipsaci Radix collected from Sichuan-1, the contents of LA, CA, LN, IA and AVI (1.10%, 8.28%, 0.61%, 0.92%, 15.19%) in crude drug were higher than those in sweated sample (0.98%, 2.49%, 0.55%, 0.70% and 2.50%); the contents of CaA, IB and IC in crude drug (0.01%, 0.09%, 0.48%) were lower than those in sweated sample (0.05%, 0.14%, 0.59%). The variations might be the results of ‘sweating’ in production area for Dipsaci Radix. Compared with the reported analytical methods of Dipsaci Radix, this newly established method provided much higher specificity, precision and accuracy. By simultaneous determination of eight major bioactive components, the quality of crude and sweated Dipsaci Radix could be controlled effectively.

### Principal component analysis

**Figure 3.**
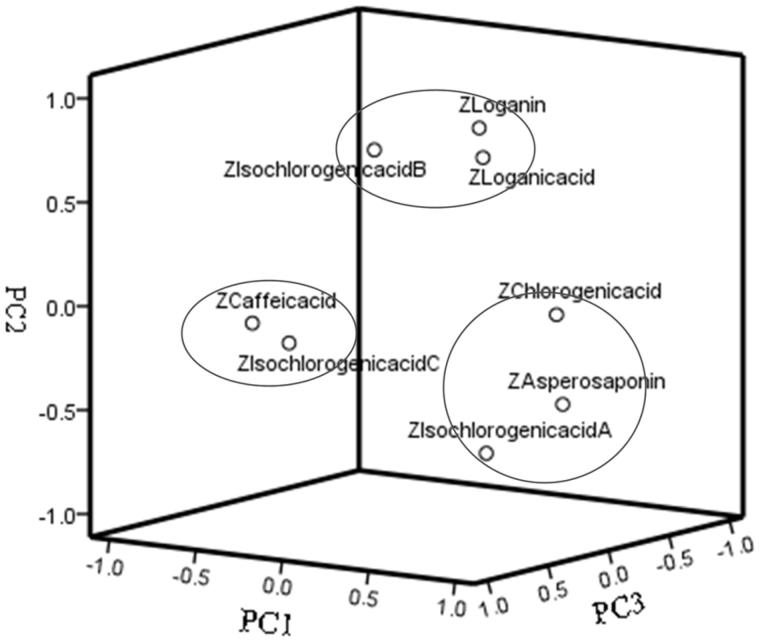
Principal component analysis (PCA) of Dipsaci Radix.

Principal component analysis (PCA) is a well-known approach to give an interpretable overview of the main information in a multivariate dataset. It could generate fewer principal components (PCs) which are independent of the original variables but show linear combinations of them, and simultaneously explain most features of the aboriginal data (Wang et al. 2012). The PCA was performed by using the contents of the eight compounds as the variances and the first 3 PCs were extracted with a cumulative contribution rate of 80.054%. The multiple regression models of each PC were obtained as follows: PC_1_ = 0.506X_1_ + 0.945X_2_ − 0.672X_3_ + 0.398X_4_ − 0.156X_5_ + 0.594X_6_ − 0.076X_7_ + 0.759X_8_; PC_2_ = 0.743X_1_ + 0.034X_2_ − 0.140X_3_ + 0.857X_4_ + 0.706X_5_ − 0.656X_6_ − 0.096X_7_ − 0.462X_8_; PC_3_ = 0.174X_1_ + 0.193X_2_ − 0.397X_3_ + 0.050X_4_ − 0.126X_5_ + 0.273X_6_ + 0.946X_7_ − 0.124X_8_, where Xi was the standardized area of common peak *i*. From the point of variance contribution rate, when eigenvalue *λ*_1_ = 2.718, PC_1_ contribution rate was 33.972%, which was the largest, and contained the most information. In three main components (Figure 3), the first principal component (PC_1_) coefficient in sequence was X_2_> X_8_> X_6_> X_1_> X_4_> X_7_> X_5_> X_3_, the coefficient represent the important degree of the corresponding compound in Dipsaci Radix Pieces quality control. The degree of importance in sequence was CA > AVI > IA > LA > LN > IC > IB > CaA.

### Discriminant analysis

To evaluate the phytochemical equivalency between crude and sweated Dipsaci Radix samples, Discriminant analysis (DA) was conducted. In this study, we used eight compounds in the crude and sweated Dipsaci Radix as a variable discriminant analysis, the contents of eight compounds were used to produce fisher linear discriminant function, and the functions were shown as below. Crude samples = −8.866X_1_ − 2.574X_2_ − 29.867X_3_ + 52.987X_4_ − 8.498X_5_ + 79.566X_6_ + 6.287X_7_ − 0.368X_8_ − 33.967. Sweated samples = −6.597X_1_ − 5.010X_2_ − 77.502X_3_ + 53.998X_4_ − 10.114X_5_ + 73.502X_6_ + 27.792X_7_ − 0.029X_8_ − 31.700.

**Figure 4.**
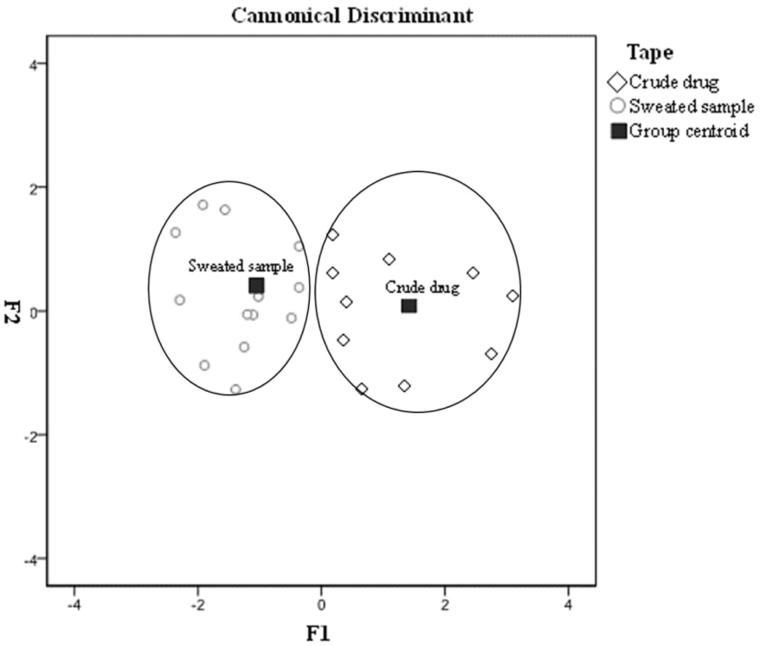
Discriminant analysis (DA) of the crude and sweated Dipsaci Radix.

As shown in Figure 4, all of the samples were clustered into two groups: sweated sample and Crude drug. DA scatter plot had a good effect for distinguishing the crude and sweated Dipsaci Radix samples.

## Conclusions

The method established in this paper was specific, accurate, and sensitive for simultaneous quantification of eight compounds in Dipsaci Radix. The method, using HPLC-DAD analysis in combination with PCA and DA, could provide a more comprehensive and quantitative chemical pattern recognition and quality evaluation to Dipsaci Radix samples. In the meantime, it provided a scientific basis for clarifying the mechanism of Dipsaci Radix sweating in the production area.
